# Prognostic significance of pre- and post-operative tumour markers for patients with gastric cancer

**DOI:** 10.1038/s41416-020-0901-z

**Published:** 2020-05-26

**Authors:** Jun-Peng Lin, Jian-Xian Lin, Yu-Bin Ma, Jian-Wei Xie, Su Yan, Jia-Bin Wang, Jun Lu, Qi-Yue Chen, Xin-Fu Ma, Long-Long Cao, Mi Lin, Ru-Hong Tu, Chao-Hui Zheng, Ping Li, Chang-Ming Huang

**Affiliations:** 1 Department of Gastric Surgery, Fujian Medical University Union Hospital, Fuzhou, Fujian Province China; 2 Key Laboratory of Ministry of Education of Gastrointestinal Cancer, Fujian Medical University, Fuzhou, Fujian Province China; 3 Department of Gastrointestinal Surgery, Affiliated Hospital of Qinghai University, Xining, Qinghai Province China

**Keywords:** Gastric cancer, Prognostic markers

## Abstract

**Background:**

In clinical practice, carcinoembryonic antigen (CEA) and carbohydrate antigen (CA) 19-9 are the most common markers measured before and after surgery for gastric cancer (GC). However, which pre- or post-operative combined tumour markers (CEA and CA19-9) have more prognostic value remains unclear.

**Methods:**

Consecutive patients undergoing a resection for GC at the Fujian Medical University Union Hospital were included as a discovery database between January 2011 and December 2014. The prognostic impact of pre- and post-operative tumour markers was evaluated using Kaplan–Meier log-rank survival analysis and multivariable Cox regression analysis. The results were then externally validated.

**Results:**

A total of 735 and 400 patients were identified in the discovery cohort and in the validation cohort, respectively. Overall survival rates decreased in a stepwise manner in association with the number of pre- and post-operative positive tumour markers (both *P* < 0.001). Multivariable analysis revealed that the number of pre-operative positive tumour markers was an independent prognostic factor (*P* < 0.05). For patients with abnormal pre-operative tumour markers, normalisation of tumour markers after surgery is an independent prognostic protective factor (hazard ratio (HR) = 0.618; 95% confidence interval (CI) = 0.414–0.921), and patients with both positive post-operative tumour markers had double the risk of overall death (HR = 2.338; 95% CI = 1.071–5.101). Similar results were observed in the internal validation and external validation cohorts.

**Conclusion:**

Pre-operative tumour markers have a better discriminatory ability for post-operative survival in GC patients than post-operative tumour markers, and the normalisation of tumour markers after surgery was associated with better survival.

## Background

Gastric cancer (GC) is the fifth most common malignancy and the third most common cause of cancer-related death worldwide.^1^ The American Joint Committee on Cancer staging system is currently recognised as reliable standard prognosticator, which does not consider prognostic determinants other than tumour, node, metastasis (TNM) stage.^2^ However, due to the differences in clinical and biological characteristics, the survival of GC with the same stage is heterogeneous. Thus, by integrating other significant prognostic factors, such as tumour markers, the individual prognosis of patients can be better assessed.^3^ In clinical practice, carcinoembryonic antigen (CEA) and carbohydrate antigen (CA) 19-9 are the most commonly used markers for the early diagnosis and monitoring of GC. These markers have been confirmed to be associated with prognosis and recurrence of GC after surgery.^4,5^ However, previous studies mainly focused on the pre-operative level of CEA and CA19-9.^6–8^ There are rare and conflicting studies evaluating the prognostic value of post-operative tumour markers.^9,10^ Therefore, the purpose of this study was to determine whether pre- or post-operative combined tumour markers (CEA and CA19-9) are more prognostic. Specifically, we asked whether patients with pre-operative positive tumour markers that normalise after gastrectomy had a better survival than patients with post-operative positive tumour markers.

## Methods

### Study population

Patients who consecutively underwent curative resection for stage I to III GC from January 2011 to December 2014 at Fujian Medical University Union Hospital (FMUUH) were enrolled in this study. As described previously,^7^ our inclusion criteria were as follows: (1) a histologically confirmed adenocarcinoma of the stomach; (2) no evidence of tumours invading the adjacent organs (pancreas, spleen, liver and transverse colon), paraaortic lymph node enlargement or distant metastasis demonstrated by abdominal computed tomography and/or abdominal ultrasound and posteroanterior chest radiographs; and (3) a D1/D1 + /D2 lymphadenectomy with a curative R0 resection. The exclusion criteria were as follows: (1) metastatic disease (*n* = 58), (2) neoadjuvant chemotherapy (*n* = 75), (3) malignant disease of other organs (*n* = 100) and (4) lack of pre- and post-operative tumour marker (CEA and CA19-9) data (*n* = 920). Finally, 735 patients were included in the study as the discovery cohort (Supplementary Fig. 1). All surgical procedures, including D2 lymphadenectomy, were performed according to the guidelines of the Japanese Gastric Cancer Association.^11^ The staging was performed according to the eighth corresponding edition of the AJCC Staging Manual.^2^ Adjuvant chemotherapy using 5-fluorouracil-based regimens (mostly oxaliplatin with either Xeloda or S1) was recommended to the majority of patients with advanced GC.^12,13^ Additional external validation was performed using a dataset from Affiliated Hospital of Qinghai University between January 2012 and December 2016, which satisfied the aforementioned inclusion and exclusion criteria. Finally, 400 patients were included as the validation cohort.

### Measurement of tumour markers

As described previously,^7^ pre-operative tumour markers were measured within 1 week before gastrectomy. Post-operative tumour markers were measured in the serum sample obtained at the first visit, between 1 and 2 months after gastrectomy.^14^ A cut-off value of 5 ng/ml was used to establish positivity for CEA, which was determined by previous study.^15,16^ The cut-off value of 37 U/ml was used to establish positivity for CA19-9.^16^ Therefore, patients were grouped according to the number of positive tumour markers as follows: (1) patients with 0 positive tumour markers (negative for both CEA and CA19-9); (2) patients with 1 positive tumour marker (either positive for CEA or CA19-9); (3) patients with 2 positive tumour markers (positive for both CEA and CA19-9). The clinical characteristics and survival rates were analysed according to the number of positive tumour markers. Patients with 0 positive tumour markers were defined as normal tumour marker group, and patients with 1 or 2 positive tumour markers were defined as abnormal tumour marker group. Additionally, the normalisation of tumour markers was defined as the change from abnormal tumour marker group before surgery to normal tumour marker group after operation.

### Follow-up investigation

Post-operative follow-up evaluations were performed every 3 months for 2 years and then every 6 months from years 3 to 5. The final follow-up evaluation was conducted in December 2018. Most routine follow-up appointments included a physical examination, laboratory testing (including CA19-9, CA72-4 and CEA measurements), chest radiography and abdominopelvic ultrasonography or computed tomography, along with an annual endoscopic examination as described previously.^17^ Overall survival (OS) was defined as the time from surgery to death from any cause or to the time of censoring on the date of the last follow-up. Cancer-specific survival (CSS) was calculated from the date of surgery to the date of death from GC.^18^

### Statistical analysis

Categorical variables were compared using the *χ*^2^ test or Fisher’s exact test, whereas differences in continuous variables between groups were analysed using the Mann–Whitney *U* test. Survival curves of different groups were estimated and compared by Kaplan–Meier method and log-rank test. The Cox proportional hazards regression model was applied to perform univariable and multivariable analyses. First, for all patients in discovery cohort (FMUUH), pre- and post-operative tumour markers with traditional clinicopathological variables were included in the univariable and multivariable analyses, and identified that pre-operative tumour markers were independent prognostic factors of survival. Second, for patients with positive tumour markers before surgery, post-operative tumour marker response and the number of positive tumour markers after surgery with traditional clinicopathological variables was included in the univariable analyses. The variables associated with survival with *P* < 0.05 and post-operative tumour marker response were included in the multivariable analysis 1. The variables associated with survival with *P* < 0.05 and the number of positive tumour markers was included in the multivariable analysis 2. The time-dependent receiver-operating characteristic (*t*-ROC) curves were used to compare the prognostic abilities of the pre- and post-operative tumour markers.^19^ Lastly, internal validation was performed by simple bootstrapping, applying resampling with replacement 10,000 times.^20^ Moreover, we validated our findings in an independent cohort. All tests were two sided with the significance level set at *P* < 0.05. All data were analysed using SPSS, ver. 22.0 (IBM Corp, Armonk, NY) and R ver. 3.5.2 (R Project). The R packages “timeROC” were used for calculation of the time-dependent ROC analysis.

## Results

### Clinicopathological characteristics

In total, 1135 patients were identified in the study. Of these patients, 735 were included in the discovery cohort and 400 in the validation cohort. For the discovery and validation cohorts, the median time to follow-up was 57.0 and 47.0 months, respectively, and the 5-year OS rates were 66.8% and 68.3%, respectively. Furthermore, the 5-year CSS of the discovery cohort and the validation cohort was 68.8% and 72.7%.

The discovery cohort comprised 569 males and 166 females with a median age of 60 years (interquartile range (IQR): 54–67 years) and a mean body mass index (BMI) of 21.7 kg/m^2^. Based on the TNM staging system, 81 (11.0%), 220 (29.9%) and 434 (59.1%) of the patients had stage I, II and III disease, respectively. Additionally, 463 (63.0%) patients received adjuvant chemotherapy after surgery. Compared with the discovery cohort, patients in the validation cohort were significantly associated with younger age, increased BMI, smaller tumour size and pathological findings, such as differentiated histological type, an earlier TNM stage, and vascular and perineural invasion (all *P* < 0.05, Supplementary Table 1). Additionally, more patients (77.8%) underwent adjuvant chemotherapy after surgery in the validation cohort (*P* < 0.001, Supplementary Table 1).

### Clinical and pathological characteristics based on pre- and post-operative tumour markers

At pre-treatment, there were 512 (69.7%) patients with no positive tumour markers, 189 (25.7%) patients with one and 34 (4.6%) with two positive tumour markers. After gastrectomy, 591 (80.4%) patients had no positive tumour markers, 131 (17.8%) patients had one and 13 (1.8%) had two positive tumour markers. Table 1 shows that an increased number of positive tumour markers before surgery was significantly associated with older age (*P* < 0.001), male sex (*P* < 0.001), proximal tumour location (*P* = 0.004), larger tumour size (*P* < 0.001) and a more advanced TNM stage (including T stage and N stage) (all *P* < 0.001). Similarly, an increased number of positive tumour markers after surgery was also associated with older age (*P* = 0.003), larger tumour size (*P* = 0.010) and a more advanced TNM stage (including T stage and N stage) (all *P* < 0.05, Table 1).

**Table 1:** Patient characteristics and associations of the number of positive tumour markers with clinicopathological findings in the discovery cohort.

Clinicopathological features	Number of positive tumour markers before surgery			*P* value^a^	Number of positive tumour markers after surgery			*P* value^a^
	0	1	2		0	1	2	
Age	58.5 ± 10.4	61.2 ± 9.9	62.7 ± 7.1	**<0.001**	58.9 ± 10.4	61.3 ± 9.6	64.8 ± 6.7	**0.003**
Sex				**<0.001**				0.15
Male	376 (73.4)	163 (86.2)	30 (88.2)		452 (76.5)	105 (80.2)	12 (92.3)	
Female	136 (26.6)	26 (13.8)	4 (11.8)		139 (23.5)	26 (19.8)	1 (7.7)	
BMI (kg/m^2^)	21.7 ± 4.3	21.9 ± 3.5	21.3 ± 4.8	0.82	21.8 ± 4.0	21.3 ± 4.9	21.8 ± 2.0	0.73
ASA score				0.20				0.94
1	331 (64.6)	127 (67.2)	26 (76.5)		388 (65.7)	88 (67.2)	8 (61.5)	
2	165 (32.2)	55 (29.1)	8 (23.5)		185 (31.3)	39 (29.8)	4 (30.8)	
3	16 (3.1)	7 (3.7)	0 (0.0)		18 (3.0)	4 (3.1)	1 (7.7)	
Tumour location				**0.004**				0.38
Upper	10 (20.5)	57 (30.2)	17 (50.0)		141 (23.9)	32 (24.4)	6 (46.2)	
Middle	136 (26.6)	47 (24.9)	5 (14.7)		149 (25.2)	37 (28.2)	2 (15.4)	
Lower	209 (40.8)	57 (30.2)	7 (20.6)		227 (38.4)	42 (32.1)	4 (30.8)	
Mixed	62 (12.1)	28 (14.8)	5 (14.7)		74 (12.5)	20 (15.3)	1 (7.7)	
Tumour size (cm)	4.6 ± 2.5	5.4 ± 2.6	6.0 ± 2.3	**<0.001**	4.7 ± 2.5	5.3 ± 2.8	6.1 ± 3.2	**0.01**
Histologic type				0.22				0.34
Differentiated	110 (21.5)	51 (27.0)	8 (23.5)		131 (22.2)	35 (26.7)	3 (23.1)	
Undifferentiated	402 (78.5)	138 (73.0)	26 (76.5)		460 (77.8)	96 (73.3)	10 (76.9)	
Vascular invasion				0.50				0.07
Negative	348 (68.0)	114 (60.3)	26 (76.5)		395 (66.8)	80 (61.1)	6 (46.2)	
Positive	164 (32.0)	75 (39.7)	8 (23.5)		196 (33.2)	51 (38.9)	7 (53.8)	
Perineural invasion				0.06				0.39
Negative	385 (75.2)	134 (70.9)	224 (83.3)		435 (73.6)	99 (75.6)	11 (84.6)	
Positive	127 (24.8)	55 (29.1)	45 (16.7)		156 (26.4)	32 (24.4)	2 (15.4)	
pT stage				**<0.001**				**0.01**
T1	63 (12.3)	10 (5.3)	1 (2.9)		60 (10.2)	13 (9.9)	1 (7.7)	
T2	72 (14.1)	14 (7.4)	1 (2.9)		7 (12.7)	11 (8.4)	1 (7.7)	
T3	205 (40.0)	79 (41.8)	13 (38.2)		251 (42.5)	43 (32.8)	3 (23.1)	
T4a	164 (32.0)	80 (42.3)	19 (55.9)		196 (33.2)	59 (45.0)	8 (61.5)	
T4b	8 (1.6)	6 (3.2)	0 (0.0)		9 (1.5)	5 (3.8)	0 (0.0)	
pN stage				**<0.001**				**<0.001**
N0	131 (25.6)	28 (14.8)	3 (8.8)		140 (23.7)	22 (16.8)	0 (0.0)	
N1	103 (20.1)	31 (16.4)	2 (5.9)		114 (19.3)	21 (16.0)	1 (7.7)	
N2	114 (22.3)	36 (19.0)	8 (23.5)		132 (22.3)	25 (19.1)	1 (7.7)	
N3a	94 (18.4)	54 (28.6)	10 (29.4)		119 (20.1)	34 (26.0)	5 (38.5)	
N3b	70 (13.7)	40 (21.2)	11 (32.4)		86 (14.6)	29 (22.1)	6 (46.2)	
pTNM stage				**<0.001**				**0.005**
I	68 (13.3)	13 (6.9)	0 (0.0)		67 11.3)	14 (10.7)	0 (0.0)	
II	176 (34.4)	38 (20.1)	6 (17.6)		192 (32.5)	26 (19.8)	2 (15.4)	
III	268 (52.3)	138 (73.0)	28 (82.4)		332 (56.2)	91 (69.5)	11 (84.6)	
Adjuvant chemotherapy				0.29				0.99
Yes	328 (64.1)	116 (61.4)	19 (55.9)		372 (62.9)	83 (63.4)	8 (61.5)	
No	184 (35.9)	73 (38.6)	15 (44.1)		219 (37.1)	48 (36.6)	5 (38.5)	

^a^Linear-by-linear association.

Statistically significant values are in bold

### Post-operative survival rates based on pre- and post-operative tumour markers

**Fig. 1:**
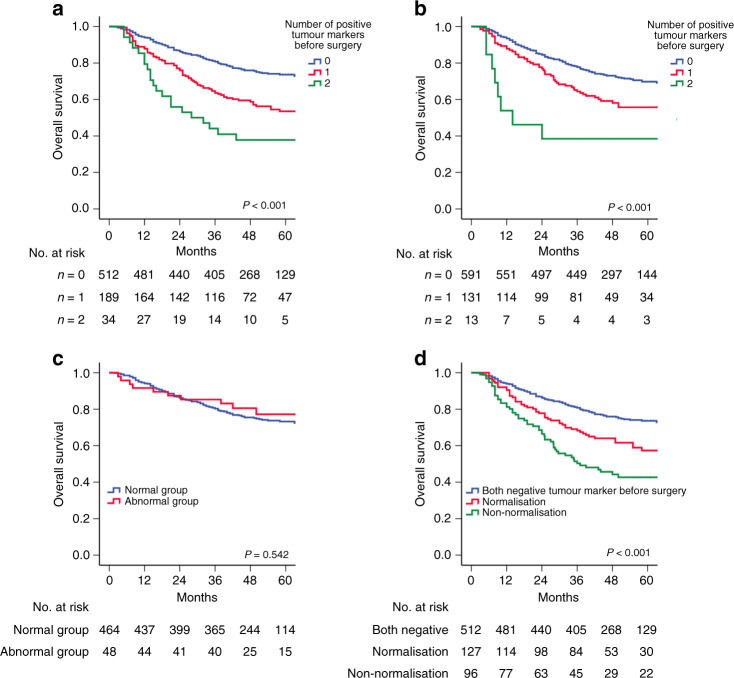
Kaplan–Meier analysis of OS of the patients who underwent curative surgery for GC. Association of the number of positive tumour markers before surgery (**a**) and after surgery (**b**) with OS of all patients who underwent curative surgery for GC. **c** OS for patients with normal and abnormal post-operative tumour markers. **d** OS for patients with normal pre-operative, normalised post-operative or non-normalised post-operative tumour markers. Figure 1c includes 512 patients with normal pre-operative tumour markers.

**Fig. 2:**
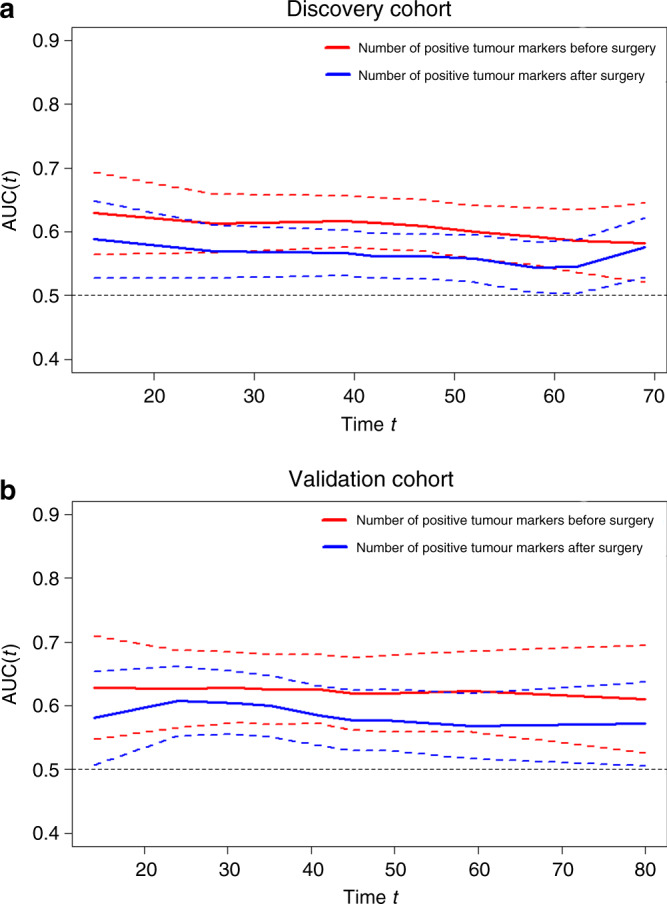
Time-dependent ROC curves for tumour makers. Time-dependent ROC curves for the number of positive tumour markers before surgery and after surgery in the discovery cohort (**a**) and validation cohort (**b**). The horizontal axis represents the year after surgery, and the vertical axis represents the estimated area under the ROC curve for survival at the time of interest. Red and blue solid lines represent the estimated AUCs of the number of positive tumour markers before surgery and after surgery, respectively, and broken lines represent the 95% confidence intervals of each AUC.

The Kaplan–Meier curves for the 5-year OS rate decreased with an increasing number of positive tumour markers before (*n* = 0: 73.6%, *n* = 1: 53.5%, *n* = 2: 37.8%, *P* < 0.001, Fig. 1a) and after surgery (*n* = 0: 69.8%, *n* = 1: 55.7%, *n* = 2: 38.5%, *P* < 0.001, Fig. 1b). In univariable analyses, the number of pre- and post-operative positive tumour markers was significantly associated with OS, as well as age, BMI, tumour location, tumour size, vascular invasion, perineural invasion, T stage, N stage and adjuvant chemotherapy (all *P* < 0.05, Table 2). In a multivariable analysis, including the above factors, the number of pre-operative positive tumour markers (*n* = 1: hazard ratio (HR) = 1.401, *P* = 0.022; *n* = 2: HR = 1.850, *P* < 0.001), but not the number of post-operative positive tumour markers (*P* > 0.05, Table 2), was an independent prognostic factor for OS. Additionally, the *t*-ROC curve of the number of pre-operative positive tumour markers was also continuously superior to that of the number of post-operative positive tumour markers throughout the observation period (Fig. 2a).

**Table 2:** Univariable and multivariable analyses of clinicopathologic variables in relation to OS in patients with GC undergoing curative resection.

Clinicopathologic characteristics	Univariable analysis		Multivariable analysis	
	HR (95% CI)	*P* value	HR (95% CI)	*P* value
Age	1.02 (1.01–1.04)	0.003	1.02 (1.01–1.00)	0.003
Sex				
Male	Reference			
Female	0.90 (0.65–1.23)	0.51		
BMI	0.93 (0.89–0.97)	0.002	0.96 (0.93–0.99)	0.01
ASA score				
1	Reference			
2	1.13 (0.86–1.47)	0.39		
3	1.29 (0.66–2.53)	0.46		
Tumour location				
Upper	Reference		–	
Middle	0.99 (0.68–1.42)	0.94	–	0.97
Lower	0.77 (0.54–1.09)	0.14	–	0.87
Mixed	1.92 (1.31–2.82)	0.001	–	0.12
Tumour size	1.02 (1.01–1.02)	<0.001	1.01 (1.00–1.01)	0.01
Histologic type				
Differentiated	Reference			
Undifferentiated	1.28 (0.92–1.78)	0.14		
Vascular invasion				
Negative	Reference		–	
Positive	2.04 (1.58–2.64)	<0.001	–	0.89
Perineural invasion				
Negative	Reference		–	
Positive	1.85 (1.41–2.42)	<0.001	–	0.14
pT stage				
T1	Reference		Reference	
T2	2.96 (0.82–10.76)	0.10	3.04 (0.83–11.09)	0.09
T3	7.58 (2.40–24.01)	0.001	4.26 (1.33–13.69)	0.02
T4a	16.87 (5.37–53.01)	<0.001	6.82 (2.11–22.01)	0.001
T4b	21.02 (5.57–79.25)	<0.001	10.31 (2.66–39.88)	0.001
pN stage				
N0	Reference		Reference	
N1	1.34 (0.71–2.53)	0.37	1.44 (0.76–2.75)	0.26
N2	2.50 (1.43–4.35)	0.001	2.16 (1.24–3.79)	0.01
N3a	5.03 (3.00–8.45)	<0.001	3.56 (2.09–6.06)	<0.001
N3b	9.79 (5.87–16.33)	<0.001	5.84 (3.25–9.93)	<0.001
Adjuvant chemotherapy				
No	Reference		Reference	
Yes	0.74 (0.57–0.96)	0.02	0.66 (0.51–0.87)	0.003
Number of positive tumour markers before surgery				
0	Reference		Reference	
1	1.98 (1.50–2.61)	<0.001	1.40 (1.05–1.87)	0.02
2	3.45 (2.17–5.47)	<0.001	1.85 (115–2.97)	<0.001
Number of positive tumour markers after surgery				
0	Reference		–	
1	1.65 (1.22–2.24)	0.001	–	0.97
2	3.73 (1.83–7.58)	<0.001	–	0.15

### Post-operative tumour marker response and survival

In the normal pre-operative tumour marker group (*n* = 512), 48 patients (9.4%) had abnormal tumour markers after surgery. The survival rate of patients with normal tumour markers after surgery was similar to that of patients with abnormal tumour markers after surgery (5-year OS: 73.3% vs. 77.2%, *P* = 0.542, Fig. 1c).

The abnormal pre-operative tumour marker group included 223 patients with a single or both positive tumour markers, of which 127 (57.0%) patients had normalised post-operative tumour markers, and 96 (43.0%) patients had positive tumour markers after surgery. The normalisation of tumour markers after surgery was only associated with N stage (*P* = 0.024), but not with other clinicopathological data (*P* > 0.05, Supplementary Table 2). The 5-year OS rate was worse in the non-normalisation group than that in the normalisation group (42.7% vs. 57.4%, *P* = 0.012), but both were worse than that of patients with normal pre-operative tumour markers (both *P* < 0.001, Fig. 1d). Multivariable analysis confirmed that normalisation of tumour markers after surgery was an independent protective factor for patients with abnormal pre-operative tumour markers (HR = 0.618, *P* = 0.018), and patients with two positive tumour markers after surgery had a more than 2-fold increased risk of overall death compared with that of patients with normalised post-operative tumour markers (HR = 2.338; *P* = 0.033, Table 3).

**Table 3:** Univariable and multivariable analyses of clinicopathologic variables in relation to OS in patients with positive tumour markers before operation.

Clinicopathologic characteristics	Univariable analysis		Multivariable analysis 1^a^		Multivariable analysis 2^a^	
	HR (95% CI)	*P* value	HR (95% CI)	*P* value	HR (95% CI)	*P* value
Age	1.03 (1.01–1.06)	0.01	1.04 (1.02–1.07)	0.001	1.04 (1.02–1.07)	0.001
Sex						
Male	Reference					
Female	1.22 (0.71–2.11)	0.48				
BMI	0.93 (0.87–0.99)	0.03	0.93 (0.88–0.98)	0.01	0.93 (0.88–0.98)	0.004
ASA score						
1	Reference					
2	1.32 (0.87–2.00)	0.20				
3	1.98 (0.80–4.92)	0.14				
Tumour location						
Upper	Reference		–		–	
Middle	0.97 (0.57–1.65)	0.91	–	0.34	–	0.40
Lower	0.82 (0.48–1.38)	0.45	–	0.77	–	0.82
Mixed	1.95 (1.15–3.33)	0.01	–	0.06	–	0.05
Tumour size	1.01 (1.01–1.02)	<0.001	1.01 (1.00–1.02)	0.02	1.01 (1.00–1.02)	0.02
Histologic type						
Differentiated	Reference					
Undifferentiated	1.08 (0.69–1.69)	0.75				
Vascular invasion						
Negative	Reference		–		–	
Positive	1.82 (1.23–2.67)	0.002	–	0.55	–	0.53
Perineural invasion						
Negative	Reference		–		–	
Positive	1.63 (1.09–2.44)	0.02	–	0.11	–	0.09
pT stage						
T1	Reference		–		–	
T2	1.69 (0.15–18.60)	0.67	–	0.72	–	0.69
T3	5.44 (0.75–39.66)	0.09	–	0.12	–	0.11
T4a	9.67 (1.34–69.82)	0.03	–	0.06	–	0.05
T4b	14.50 (1.62–129.96)	0.02	–	0.27	–	0.25
pN stage						
N0	Reference		Reference		Reference	
N1	2.12 (0.73–6.21)	0.17	2.20 (0.75–6.45)	0.15	2.17 (0.74–6.37)	0.16
N2	3.36 (1.25–9.00)	0.02	4.14 (1.53–11.17)	0.01	4.08 (1.51–11.01)	0.01
N3a	3.87 (1.51–9.96)	0.01	4.00 (1.54–10.35)	0.004	3.85 (1.48–10.00)	0.01
N3b	8.61 (3.37–21.95)	<0.001	7.88 (3.03–20.49)	<0.001	7.63 (2.93–19.89)	<0.001
Adjuvant chemotherapy						
No	Reference		Reference		Reference	
Yes	0.59 (0.40–0.88)	0.01	0.56 (0.37–0.84)	0.01	0.56 (0.37–0.84)	0.01
Post-operative tumour marker response						
Non-normalisation	Reference		Reference			
Normalisation	0.61 (0.42–0.90)	0.01	0.62 (0.41–0.92)	0.02		
Number of positive tumour markers after surgery						
0	Reference				Reference	
1	1.54 (1.03–2.30)	0.04			1.54 (1.02–2.34)	0.04
2	2.50 (1.18–5.28)	0.02			2.34 (1.07–5.10)	0.03

^a^Multivariable analysis 1 included post-operative tumour markers response, excluding the number of positive tumour markers after treatment.

^b^Multivariable analysis 2 included the number of positive tumour markers after treatment, excluding post-operative tumour markers response.

### Internal validation

In the discovery cohort, internal validation identified that the number of pre-operative positive tumour markers (*n* = 1: HR = 1.399, *P* = 0.034; *n* = 2: HR = 1.736, *P* < 0.001), but not the number of post-operative positive tumour markers (*P* > 0.05, Supplementary Table 3), was an independent prognostic factor for OS.

For patients with abnormal tumour markers, internal validation revealed that normalisation of tumour markers after surgery was an independent protective factor (HR = 0.636, *P* = 0.032, Supplementary Table 4), and patients with both positive tumour markers after surgery had a more than 2-fold increased risk of overall death compared with patients with normalised post-operative tumour markers (HR = 2.909; *P* = 0.010, Supplementary Table 4).

### External validation

In the validation cohort, there were 300 (75.0%) patients with no positive tumour markers before surgery, 85 (21.3%) patients with one, and 15 (3.8%) with two positive tumour markers before surgery. After the operation, 344 (86.0%) patients had no positive tumour markers, 49 (12.3%) patients had one and 7 (1.8%) had two positive tumour markers. In the univariable analysis, an increase in the number of pre- and post-operative positive tumour markers was associated with shorter OS (both *P* < 0.05, Supplementary Table 5). Multivariable analyses revealed that only the number of pre-operative positive tumour markers was independently associated with shorter OS (1: HR = 1.628, *P* = 0.019; 2: HR = 2.289, *P* = 0.023, Supplementary Table 5).

The abnormal pre-operative tumour marker group included 100 patients, of whom 64 (64.0%) had normalised post-operative tumour markers and 36 (36.0%) had non-normalised post-operative tumour markers. Multivariable analysis revealed that normalisation of tumour markers after surgery was an independent prognostic factor for OS (HR = 0.422, *P* = 0.002, Supplementary Table 6). Similarly, the mortality risk of patients with both positive post-operative tumour markers was significantly higher than that of patients with both negative post-operative tumour markers (HR = 3.034, *P* = 0.017, Supplementary Table 6).

## Discussion

As common indicators of early pre-operative diagnosis and routine monitoring of post-operative follow-up of cancer, the prognostic values of serum CEA and CA19-9 for GC remain under investigation, and most previous studies only focus on the levels of tumour markers before surgery.^21,22^ In particular, there are few studies on the prognostic effects of post-operative CEA and CA19-9. Toyoda et al.^23^ found that the combination of multiple tumour markers can improve the prediction of survival compared with a single tumour marker. Therefore, our study assessed the prognostic value of the combination of CEA and CA19-9 before and after surgery for GC by multi-centre data. All patients were grouped according to the number of positive tumour markers. CEA and CA19-9 have been reported to correlate with disease burden.^7,24,25^ In this study, an increase in tumour size and pathological stage were associated with the number of positive pre-operative tumour markers, as in our previous report.^7^ Additionally, similar results were found for the post-operative tumour markers, which indicated that both pre- and post-operative tumour markers may be associated with poor prognosis of GC.

At present, it is still controversial whether pre- or post-operative tumour markers can better predict the prognosis of GC. Uda et al.^10^ found that the predictive performance of the pre-operative levels of tumour markers was superior compared with that of the post-operative values. However, Suenaga et al.^9^ suggested that post-operative CEA and CA19-9 have better prognostic value for patients with stage II/III GC. In this study, we firstly found that the prognostic value of pre-operative tumour markers was better than that of post-operative tumour markers, and verified it externally. Furthermore, the later the stage, the higher the level of pre-operative tumour markers (Supplementary Fig. 2). However, there are only 74 and 64 patients with early stage in the discovery cohort and validation cohort, respectively. Limited by the small number of early-stage patients, we cannot carry out further stratified analysis according to the stage. Park et al.^26^ found that post-operative serum CEA surveillance is used most effectively for colorectal cancer when patients have high pre-operative serum CEA levels. Therefore, according to the status of pre-operative tumour markers, we divided all of the patients into a normal group and an abnormal group. For patients with normal pre-operative tumour markers, only 9.4% had positive tumour markers after surgery, and the status of post-operative tumour markers was not associated with OS. However, for patients with abnormal pre-operative tumour markers, 57.0% had non-normalised tumour markers after surgery. Although the prognosis of patients in the normalisation group was better than that in the non-normalisation group, both were worse than that of patients with normal pre-operative tumour markers, which indicated that pre-operative tumour markers had a better discriminatory ability for OS.

The elevation of tumour markers after surgery may indicate residual minute cancer cells that cannot be identified during surgery and imaging examination after treatment.^27^ Our study showed that the prognosis of patients with normalised post-operative tumour markers was significantly better than that of patients with non-normalised post-operative tumour markers, which was consistent with previous studies.^14^ Furthermore, in the abnormal pre-operative tumour marker group, the number of post-operative positive tumour markers was an independent prognostic factor, and the post-operative mortality of patients positive for both CEA and CA19-9 after surgery was more than twice that of patients with normal post-operative tumour markers. Thus, these patients may benefit from multidisciplinary therapies and need timely follow-up to improve long-term survival. Since this study is a single-centre study, our results need further appropriate internal and external validation.^28^ In this study, we used the bootstrap resampling method for internal validation, and external validation was performed using an independent centre. Similar results were obtained with both the internal and external validation, which further confirmed that the predictive performance of pre-operative tumour markers is better than that of post-operative tumour markers, and normalisation of tumour markers after operation is associated with better survival for patients with GC.

Along with the rapid advances in next-generation sequencing technology, the molecular nosology of gastro-oesophageal cancers is now better understood. In addition to CEA and CA19-9, circulating tumour DNA (ctDNA) has been widely concerned in liquid biopsies. Recently, many studies have investigated the use of ctDNA for prognostic value in gastrointestinal cancers.^29,30^ However, due to the lack of DNA data, we cannot compare the prognostic value of ctDNA with tumour makers, which need further study to evaluated.

This study has several limitations. First, as a retrospective study, it may have been subject to selection bias. For example, patients who had post-operative tumour markers measured were more likely to have stage III disease (59.9% vs. 46.9%, *P* < 0.05), suggesting that patients with a higher risk had post-operative tumour marker testing. The timing of post-operative tumour marker measurement was not controlled, although it was limited to between 1 and 2 months after surgery. Second, there were significant differences of clinicopathological features between discovery and validation cohorts. However, the prognostic values of tumour markers were consistent in the two cohorts. Lastly, in the early period, CA72-4 was not the recommended tumour marker for routine testing pre-operatively in China.^31^ Therefore, the data of CA72-4 in our study are incomplete, which may affect the prognosis of GC. Despite these limitations, our study demonstrated that the prognostic value of pre-operative CEA and CA19-9 exceeds that of post-operative CEA and CA19-9 and validates our findings externally in an independent cohort. Routine measurement of post-operative tumour markers is warranted for patients with pre-operative positive tumour markers.

## Supplementary information

Supplementary files

## Data Availability

The datasets used and/or analysed during the current study are available from the corresponding author on reasonable request.
