# Supplementary material for: Prognostic significance of pre- and post-operative tumour markers for patients with gastric cancer
Source: Br J Cancer. 2020 May 26;123(3):418–25. doi: 10.1038/s41416-020-0901-z (PMC7403417; doi:10.1038/s41416-020-0901-z)
Supplement: Supplementary file 1 — Supplementary files [file 41416_2020_901_MOESM1_ESM.doc]

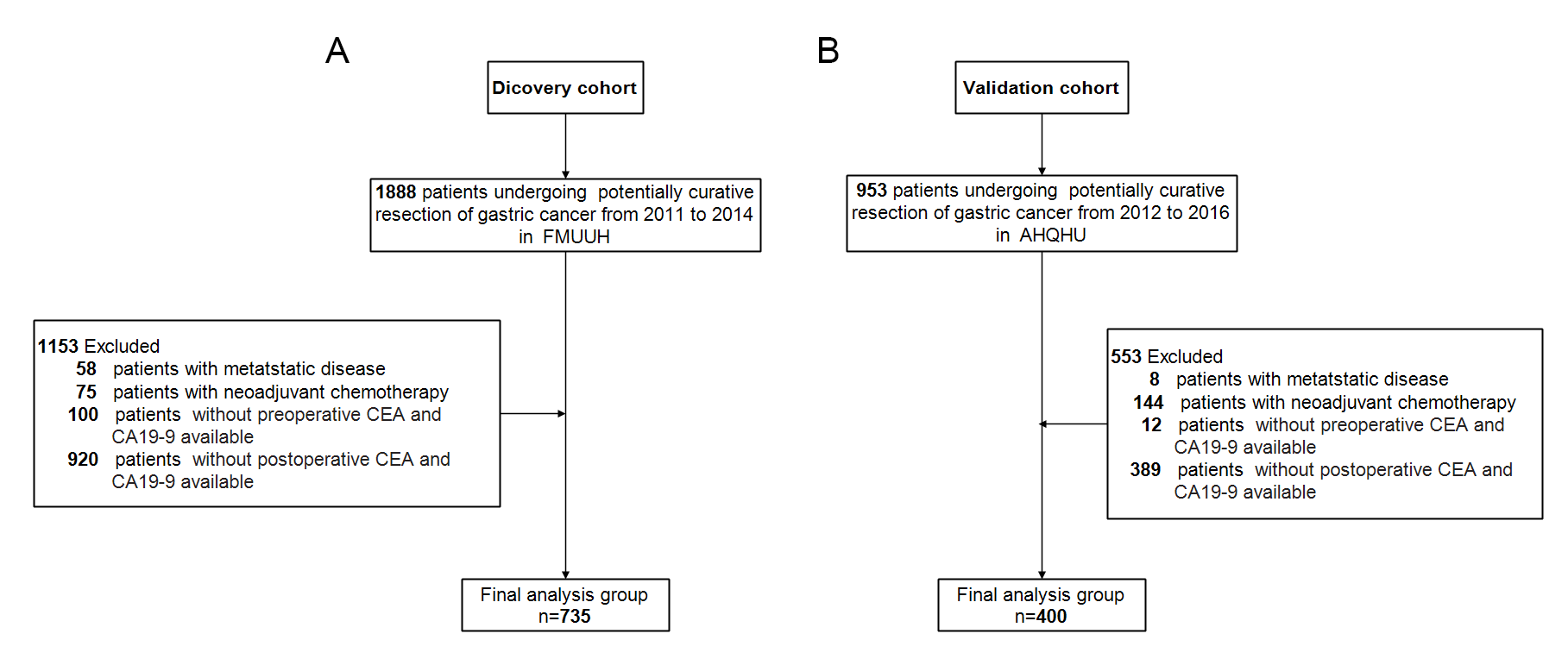


**Supplemental Figure 1.** Diagram representing the selection of the study population.

(A). Discovery cohort. (B). Validation cohort.

FMUUH, Fujian Medical University Union Hospital; AHQHU, Affiliated Hospital of Qinghai University.





**Supplemental Figure 2.** The association between the level of preoperative tumor makers and tumor stage.

Supplemental Table 1. Baseline Patient Clinicopathologic Characteristics.

| Clinicopathological Features | Discovery Cohort (n=735) (%) | Validation Cohort (n=400) (%) | P |
| --- | --- | --- | --- |
|
| Age, mean±SD | 59.4±10.2 | 56.4±10.2 | <0.001 |
| Sex |  |  | 0.73 |
| Male | 569(77.4) | 569(77.4) |  |
| Female | 166(22.6) | 166(22.6) |  |
| BMI (kg/m2)*, mean±SD | 21.7±4.2 | 22.7±3.5 | <0.001 |
| ASA score |  |  | NA |
| 1 | 484(65.9) | NA |  |
| 2 | 228(31.0) | NA |  |
| 3 | 23(3.1) | NA |  |
| Tumor Location |  |  | <0.001 |
| Upper | 179(24.4) | 102(25.5) |  |
| Middle | 188(25.6) | 143(35.8) |  |
| Lower | 273(37.1) | 146(36.5) |  |
| Mixed | 95(12.9) | 9(2.3) |  |
| Tumor size (cm), mean±SD | 4.9±2.6 | 4.1±2.1 | <0.001 |
| Histologic type |  |  | 0.01 |
| Differentiated | 169(23.0) | 121(30.3) |  |
| Undifferentiated | 566(77.0) | 279(69.8) |  |
| Vascular invasion |  |  | <0.001 |
| Negative | 481(65.4) | 126(31.5) |  |
| Positive | 254(34.6) | 217(54.3) |  |
| Unknown | 0(0.0) | 57(14.2) |  |
| Perineural invasion |  |  | <0.001 |
| Negative | 545(74.1) | 147(36.8) |  |
| Positive | 190(25.9) | 196(49.0) |  |
| Unknown | 0(0.0) | 57(14.2) |  |
| pT stage |  |  | <0.001 |
| T1 | 74(10.1) | 64(16.0) |  |
| T2 | 87(11.8) | 123(30.8) |  |
| T3 | 297(40.4) | 48(12.0) |  |
| T4a | 263(35.8) | 147(36.8) |  |
| T4b | 14(1.9) | 18(4.5) |  |
| pN stage |  |  | <0.001 |
| N0 | 162(22.0) | 188(47.1) |  |
| N1 | 136(18.5) | 70(17.5) |  |
| N2 | 158(21.5) | 52(13.0) |  |
| N3a | 158(21.5) | 64(16.0) |  |
| N3b | 121(16.5) | 25(6.3) |  |
| pTNM stage |  |  | <0.001 |
| I | 81(11.0) | 130(32.5) |  |
| II | 220(29.9) | 115(28.7) |  |
| III | 434(59.0) | 155(38.8) |  |
| Adjuvant chemotherapy |  |  | <0.001 |
| Yes | 463(63.0) | 311(77.8) |  |
| No | 272(37.0) | 89(22.3) |  |
| Number of positive tumor markers before surgery |  |  | 0.16 |
| 0 | 512(69.7) | 300(75.0) |  |
| 1 | 189(25.7) | 85(21.3) |  |
| 2 | 34(4.6) | 15(3.8) |  |
| Number of positive tumor markers after surgery |  |  | 0.05 |
| 0 | 591(80.4) | 344(86.0) |  |
| 1 | 131(17.8) | 49(12.3) |  |
| 2 | 13(1.8) | 7(1.8) |  |

*287 patients with BMI data in validation cohort.

NA: No correlated data

Supplemental Table 2. The Associations of Postoperative Tumor Markers with Clinicopathological Features

| Clinicopathological Features | Normalization (n=127) | Non-normalization (n=96) | P |
| --- | --- | --- | --- |
| Age | 61.3±9.3 | 61.7±9.8 | 0.77 |
| Sex |  |  | 0.67 |
| Male | 111(87.4) | 82(85.4) |  |
| Female | 16(12.6) | 14(14.6) |  |
| BMI (kg/m2) | 21.9±3.7 | 21.8±3.8 | 0.85 |
| ASA score |  |  | 0.14 |
| 1 | 91(71.7) | 62(64.6) |  |
| 2 | 34(26.8) | 29(30.2) |  |
| 3 | 2(1.6) | 5(5.2) |  |
| Tumor Location |  |  | 0.37 |
| Upper | 42(33.1) | 32(33.3) |  |
| Middle | 23(18.1) | 29(30.2) |  |
| Lower | 43(33.9) | 21(21.9) |  |
| Mixed | 19(15.0) | 14(14.6) |  |
| Tumor size (cm) | 5.4±2.5 | 5.7±2.6 | 0.27 |
| Histologic type |  |  | 0.46 |
| Differentiated | 36(28.3) | 23(24.0) |  |
| Undifferentiated | 91(71.7) | 73(76.0) |  |
| Vascular invasion |  |  | 0.15 |
| Negative | 81(63.8) | 52(54.2) |  |
| Positive | 46(36.2) | 44(45.8) |  |
| Perineural invasion |  |  | 0.74 |
| Negative | 90(70.9) | 70(72.9) |  |
| Positive | 37(29.1) | 26(27.1) |  |
| pT stage |  |  | 0.73 |
| T1 | 5(3.9) | 6(6.3) |  |
| T2 | 7(5.5) | 8(8.3) |  |
| T3 | 61(48.0) | 31(32.3) |  |
| T4a | 51(40.2) | 48(50.0) |  |
| T4b | 3(2.4) | 3(3.1) |  |
| pN stage |  |  | 0.02 |
| N0 | 20(15.7) | 11(11.5) |  |
| N1 | 20(15.7) | 13(13.5) |  |
| N2 | 32(25.2) | 12(12.5) |  |
| N3a | 32(25.2) | 32(33.3) |  |
| N3b | 23(18.1) | 28(29.2) |  |
| pTNM stage |  |  | 0.79 |
| I | 6(4.7) | 7(7.3) |  |
| II | 29(22.8) | 15(15.6) |  |
| III | 92(72.4) | 74(77.1) |  |
| Adjuvant chemotherapy |  |  | 0.97 |
| Yes | 77(60.6) | 58(60.4) |  |
| No | 58(39.4) | 38(39.6) |  |

Supplemental Table 3. Internal validation of clinicopathologic variables in relation to OS.

| Clinicopathologic Characteristics | Internal validation | |
| --- | --- | --- |
|  | HR (95% CI) | P* |
| Age | 1.02(1.01-1.04) | <0.001 |
| BMI | 0.94(0.90-0.98) | 0.01 |
| Tumor size | 1.01(1.00-1.01) | 0.03 |
| pT stage |  |  |
| T1 | Reference |  |
| T2 | 1.98(0.68-5.78) | 0.26 |
| T3 | 2.82(1.13-7.00) | 0.04 |
| T4a | 4.43(1.75-11.17) | 0.003 |
| T4b | 6.86(2.19-21.47) | 0.002 |
| pN stage |  |  |
| N0 | Reference |  |
| N1 | 1.26(0.76-2.07) | 0.53 |
| N2 | 1.95(1.22-3.11) | 0.02 |
| N3a | 3.21(1.98-5.22) | <0.001 |
| N3b | 5.16(3.18-8.37) | <0.001 |
| Adjuvant chemotherapy |  |  |
| No | Reference |  |
| Yes | 0.63(0.48-0.83) | <0.001 |
| Number of positive tumor markers before surgery |  |  |
| 0 | Reference |  |
| 1 | 1.40(1.06-1.84) | 0.03 |
| 2 | 1.74(1.10-2.88) | <0.001 |
| Number of positive tumor markers after surgery |  |  |
| 0 | — |  |
| 1 | — | 0.61 |
| 2 | — | 0.10 |

*Adjusted for the following variables: age, BMI, tumor location, vascular invasion, perineural invasion, pT stage, pN stage, and adjuvant chemotherapy.

Supplemental Table 4. Internal validation of clinicopathologic variables in relation to OS of patients with positive tumor markers before operation.

| Clinicopathologic Characteristics | Internal validation 1 | |  | Internal validation 2 | |
| --- | --- | --- | --- | --- | --- |
|  | HR (95% CI) | P* |  | HR (95% CI) | P* |
| Age | 1.04(1.03-1.05) | 0.002 |  | 1.04(1.03-1.05) | 0.003 |
| BMI | 0.91(0.88-0.94) | 0.01 |  | 0.91(0.88-0.94) | 0.01 |
| Tumor size | 1.00(1.00-1.01) | 0.04 |  | 1.00(1.00-1.01) | 0.04 |
| pN stage |  |  |  |  |  |
| N0 | Reference |  |  | Reference |  |
| N1 | 2.06(1.29-3.29) | 0.05 |  | 2.03(1.24-3.04) | 0.05 |
| N2 | 3.00(1.93-4.67) | 0.03 |  | 2.83(1.81-4.42) | 0.04 |
| N3a | 2.93(1.91-4.51) | 0.03 |  | 2.73(1.79-4.15) | 0.04 |
| N3b | 5.73(3.75-8.75) | <0.001 |  | 5.36(3.46-8.30) | <0.001 |
| Adjuvant chemotherapy |  |  |  |  |  |
| No | Reference |  |  | Reference |  |
| Yes | 0.49(0.39-0.61) | 0.001 |  | 0.48(0.38-0.61) | <0.001 |
| Postoperative tumor markers |  |  |  |  |  |
| Non-normalization | Reference |  |  |  |  |
| Normalization | 0.64(0.52-0.79) | 0.03 |  |  |  |
| Number of positive tumor markers after surgery |  |  |  |  |  |
| 0 |  |  |  | Reference |  |
| 1 |  |  |  | 1.48(1.21-1.80) | 0.04 |
| 2 |  |  |  | 2.91(1.94-4.36) | 0.01 |

Internal validation 1 includes postoperative tumor markers.

Internal validation 2 includes the number of positive tumor markers after treatment.

*Adjusted for the following variables: age, BMI, tumor location, vascular invasion, perineural invasion, pT stage, pN stage, and adjuvant chemotherapy.

**Supplemental Table 5. Univariable and multivariable analyses of clinicopathologic variables in relation to OS in patients with GC undergoing curative resection in the external validation cohort.**

| Clinicopathologic Characteristics | Univariable Analysis | |  | Multivariable Analysis | |
| --- | --- | --- | --- | --- | --- |
|  | HR (95% CI) | P |  | HR (95% CI) | P |
| Age | 1.03(1.01-1.05) | 0.01 |  | 1.02(1.00-1.04) | 0.02 |
| Sex |  |  |  |  |  |
| Male | Reference |  |  |  |  |
| Female | 0.72(0.48-1.14) | 0.17 |  |  |  |
| BMI* | 0.98(0.93-1.04) | 0.59 |  |  |  |
| Tumor Location |  |  |  |  |  |
| Upper | Reference |  |  |  |  |
| Middle | 0.90(0.58-1.41) | 0.64 |  |  |  |
| Lower | 0.76(0.48-1.21) | 0.24 |  |  |  |
| Mixed | 0.30(0.04-2.22) | 0.24 |  |  |  |
| Tumor size | 1.15(1.07-1.24) | <0.001 |  | — | 0.69 |
| Histologic type |  |  |  |  |  |
| Differentiated | Reference |  |  | Reference |  |
| Undifferentiated | 1.67(1.08-2.61) | 0.02 |  | 1.59(1.02-2.48) | 0.04 |
| Vascular invasion |  |  |  |  |  |
| Negative | Reference |  |  | — |  |
| Positive | 2.83(1.76-4.55) | <0.001 |  | — | 0.09 |
| Unknown | 0.70(0.30-1.66) | 0.42 |  | — | 0.33 |
| Perineural invasion |  |  |  |  |  |
| Negative | Reference |  |  | — |  |
| Positive | 2.87(1.83-4.48) | <0.001 |  | — | 0.02 |
| Unknown | 0.78(0.35-1.74) | 0.55 |  | — | 0.54 |
| pT stage |  |  |  |  |  |
| T1 | Reference |  |  | — |  |
| T2 | 1.96(0.78-4.96) | 0.15 |  | — | 0.11 |
| T3 | 6.30(2.48-15.98) | <0.001 |  | — | 0.12 |
| T4a | 5.49(2.31-13.04) | <0.001 |  | — | 0.15 |
| T4b | 10.57(3.82-29.23) | <0.001 |  | — | 0.80 |
| pN stage |  |  |  |  |  |
| N0 | Reference |  |  | Reference |  |
| N1 | 3.33(1.87-5.95) | <0.001 |  | 2.91(1.62-5.23) | <0.001 |
| N2 | 3.39(1.81-6.32) | <0.001 |  | 3.05(1.63-5.72) | 0.001 |
| N3a | 5.98(3.49-10.25) | <0.001 |  | 5.04(2.91-8.74) | <0.001 |
| N3b | 13.42(7.22-24.93) | <0.001 |  | 10.63(5.57-20.29) | <0.001 |
| Adjuvant chemotherapy |  |  |  |  |  |
| No | Reference |  |  |  |  |
| Yes | 1.31(0.81-2.09) | 0.27 |  |  |  |
| Number of positive tumor markers before surgery |  |  |  |  |  |
| 0 | Reference |  |  | Reference |  |
| 1 | 2.64(1.79-3.89) | <0.001 |  | 1.63(1.08-2.45) | 0.02 |
| 2 | 3.31(1.65-6.64) | 0.001 |  | 2.29(1.12-4.68) | 0.02 |
| Number of positive tumor markers after surgery |  |  |  |  |  |
| 0 | Reference |  |  | — |  |
| 1 | 2.59(1.65-4.04) | <0.001 |  | — | 0.82 |
| 2 | 4.01(1.62-9.89) | 0.003 |  | — | 0.12 |

*****For patients with BMI data.

Supplemental Table 6. Univariable and multivariable analyses of clinicopathologic variables in relation to OS of patients with positive tumor markers before operation in the external validation cohort.

| Clinicopathologic Characteristics | Univariable Analysis | |  | Multivariable Analysis 1* | |  | Multivariable Analysis 2* | |
| --- | --- | --- | --- | --- | --- | --- | --- | --- |
|  | HR (95% CI) | P |  | HR (95% CI) | P |  | HR (95% CI) | P |
| Age | 1.02(0.98-1.05) | 0.35 |  |  |  |  |  |  |
| Sex |  |  |  |  |  |  |  |  |
| Male | Reference |  |  |  |  |  |  |  |
| Female | 0.63(0.29-1.33) | 0.22 |  |  |  |  |  |  |
| BMI^#^ | 1.09(1.0-1.18) | 0.06 |  |  |  |  |  |  |
| Tumor Location |  |  |  |  |  |  |  |  |
| Upper | Reference |  |  |  |  |  |  |  |
| Middle | 0.89(0.47-1.70) | 0.72 |  |  |  |  |  |  |
| Lower | 0.61(0.30-1.24) | 0.17 |  |  |  |  |  |  |
| Mixed | NA | NA |  |  |  |  |  |  |
| Tumor size | 1.08(0.96-1.22) | 0.20 |  |  |  |  |  |  |
| Histologic type |  |  |  |  |  |  |  |  |
| Differentiated | Reference |  |  |  |  |  |  |  |
| Undifferentiated | 0.91(0.51-1.61) | 0.75 |  |  |  |  |  |  |
| Vascular invasion |  |  |  |  |  |  |  |  |
| Negative | Reference |  |  | Reference |  |  | Reference |  |
| Positive | 2.28(1.14-4.56) | 0.02 |  | 2.33(1.17-4.67) | 0.02 |  | 2.24(1.11-4.55) | 0.03 |
| Unknown | 0.44(0.06-3.46) | 0.44 |  | 0.40(0.05-3.10) | 0.38 |  | 0.40(0.05-3.13) | 0.38 |
| Perineural invasion |  |  |  |  |  |  |  |  |
| Negative | Reference |  |  | — |  |  | — |  |
| Positive | 2.30(1.18-4.49) | 0.02 |  | — | 0.30 |  | — | 0.28 |
| Unknown | 0.52(0.07-4.03) | 0.53 |  | — | 0.75 |  | — | 0.74 |
| pT stage |  |  |  |  |  |  |  |  |
| T1 | Reference |  |  |  |  |  |  |  |
| T2 | 1.55(0.19-12.60) | 0.68 |  |  |  |  |  |  |
| T3 | 3.40(0.43-26.85) | 0.25 |  |  |  |  |  |  |
| T4a | 3.91(0.53-28.68) | 0.18 |  |  |  |  |  |  |
| T4b | 6.25(0.75-52.12) | 0.09 |  |  |  |  |  |  |
| pN stage |  |  |  |  |  |  |  |  |
| N0 | Reference |  |  | — |  |  | — |  |
| N1 | 1.75(0.66-4.69) | 0.26 |  | — | 0.79 |  | — | 0.84 |
| N2 | 2.27(0.88-5.89) | 0.09 |  | — | 0.94 |  | — | 0.98 |
| N3a | 3.16(1.36-7.34) | 0.01 |  | — | 0.53 |  | — | 0.55 |
| N3b | 4.86(1.91-12.68) | 0.001 |  | — | 0.55 |  | — | 0.43 |
| Adjuvant chemotherapy |  |  |  |  |  |  |  |  |
| No | Reference |  |  |  |  |  |  |  |
| Yes | 1.07(0.46-2.51) | 0.88 |  |  |  |  |  |  |
| Postoperative tumor marker response |  |  |  |  |  |  |  |  |
| Non-normalization | Reference |  |  | Reference |  |  |  |  |
| Normalization | 0.45(0.26-0.77) | 0.004 |  | 0.42(0.24-0.73) | 0.002 |  |  |  |
| Number of positive tumor markers after surgery |  |  |  |  |  |  |  |  |
| 0 | Reference |  |  |  |  |  | Reference |  |
| 1 | 2.00(1.11-3.59) | 0.02 |  |  |  |  | 2.23(1.24-4.02) | 0.01 |
| 2 | 3.90(1.58-9.62) | 0.003 |  |  |  |  | 3.03(1.22-7.56) | 0.02 |

*Multivariable analysis 1 included postoperative tumor markers response, excluding the number of positive tumor markers after treatment.

*Multivariable analysis 2 included the number of positive tumor markers after treatment, excluding postoperative tumor markers response.

#For patients with BMI data.

NA: only 1 individual with mixed tumor location
